# Pro-Inflammatory Responses in Human Bronchial Epithelial Cells Induced by Spores and Hyphal Fragments of Common Damp Indoor Molds

**DOI:** 10.3390/ijerph16061085

**Published:** 2019-03-26

**Authors:** Elisabeth Øya, Rune Becher, Leni Ekeren, Anani K.J. Afanou, Johan Øvrevik, Jørn A. Holme

**Affiliations:** 1 Department of Air Pollution and Noise, Division of Infection Control and Environment and Health, Norwegian Institute of Public Health, P.O. Box 4404 Nydalen, N-0403 Oslo, Norway; Rune.Becher@fhi.no (R.B.); Leni.Ekeren@fhi.no (L.E.); Johan.Ovrevik@fhi.no (J.Ø.); JornAndreas.Holme@fhi.no (J.A.H.); 2 Department for the Chemical and Biological Work Environment, National Institute of Occupational Health, N-0403 Oslo, Norway; Anani.Afanou@stami.no; 3 Department of Biosciences, Faculty of Mathematics and Natural Sciences, University of Oslo, N-0315 Oslo, Norway

**Keywords:** mold, inflammation, lung cells, cytokines, TLRs

## Abstract

Damp indoor environments contaminated with different mold species may contribute to the development and exacerbation of respiratory illnesses. Human bronchial epithelial BEAS-2B cells were exposed to X-ray treated spores and hyphal fragments from pure cultures of *Aspergillus fumigatus*, *Penicillum chrysogenum*, *Aspergillus versicolor* and *Stachybotrys chartarum*. Hyphal fragments of *A. fumigatus* and *P. chrysogenum* induced expression and release of the pro-inflammatory cytokine interleukin (IL)-6 and the chemokine IL-8, while none of the other hyphal preparations had effects. Hyphal fragments from *A. fumigatus* and *P. chrysogenum* also increased the expression of IL-1α, IL-1β and tumor necrosis factor (TNF)-α, but these cytokines were not released. X-ray treated spores had little or no inflammatory potential. Attenuating Toll-like receptor (TLR)-2 by blocking antibodies strongly reduced the *A. fumigatus* and *P. chrysogenum* hyphae-induced IL-6 and IL-8 release, whereas TLR4 antagonist treatment was without effects. Untreated *A. fumigatus* spores formed hyphae and triggered expression of pro-inflammatory genes with similarities to the effects of hyphal fragments. In conclusion, while X-ray treated spores induced no pro-inflammatory responses, hyphal fragments of *A. fumigatus* and *P. chrysogenum* enhanced a TLR2-dependent expression and release of IL-6 and IL-8.

## 1. Introduction

Dampness and moldy indoor environments are associated with adverse respiratory diseases, including allergic and infectious conditions with enhanced inflammatory state [1,2,3]. Recently fungal dysbiosis occurring on highly exposed biological barrier surfaces including the pulmonary airways have been suggested to have a role in shaping the immune responses [4].

*Aspergillus fumigatus*, *Penicillium chrysogenum*, *Aspergillus versicolor* and *Stachybotrys chartarum* are species that commonly occur in water-damaged indoor environments [5]. Both spores and hyphal fragments are liberated from cultures of molds and may become airborne [6,7,8,9]. When inhaled, they may germinate and form hyphae [10]. While *A. fumigatus* is pathogenic [11,12,13], *P. chrysogenum* is rarely responsible for invasive disease [14,15].

Upon inhalation, spores may adhere to airway cells such as respiratory epithelial cells [16,17]. If internalized by the epithelial cells they may escape from immune cells and germinate which causes damage to the cells, while others are transported to lysosomes and destroyed [18,19,20]. However, in healthy individuals with functional immune systems and normal lungs, spores and hyphal fragments are most often trapped in the mucus lining at the epithelial surfaces of the airways and removed by mucociliary clearance, or rapidly phagocytized and eliminated by resident alveolar macrophages [21,22].

Inflammatory reactions in the lung are considered to be central in the development and exacerbation of mold-induced health effects [17,23]. However, the impact of various mold species with regard to respiratory diseases associated with inflammatory effects has not been fully elucidated. Notably, the relative levels of *A. fumigatus* in indoor air are low compared to *P. chrysogenum* [24] which is highly predominant in indoor air [14,15]. Pro-inflammatory responses are central key elements for reducing the chances of infection due to exposure to bacteria as well as mold [10]. On the other hand, chronic low-grade inflammation from continuous exposure to molds or other biological components may contribute to development or exacerbation of respiratory diseases. Lung epithelial cells represent the primary barrier preventing entry of inhaled compounds, and they play key roles in pulmonary host defense, including inflammation and regulation of immune responses [25]. However, while several studies have investigated the effects of molds on various immune cells, few have focused on the interactions between molds and lung epithelial cells [16,17,18,19,20,21,22,23,24,25,26].

So far no clear association has been documented between the total occurrence of fungal spores in air and adverse health effects in indoor environments [1]. Thus, there is a need to more specifically elucidate the pro-inflammatory potential of both aerosolized spores and hyphal fragme–nts from different species using various experimental models, in order to better understand and explain the strong association found between dampness and moldy indoor environments and adverse health effects [3,27].

Spores forming hyphae, various extracts and/or specific components from mold are often used to study mechanisms regarding mold recognition, inflammatory responses and “killing”. Studies have found that the Toll-like (TLRs), NOD-like (NLRs), C-type lectin-like (CLRs) and protease-activated receptors (PARs) recognize both spores and hyphae, suggesting a role for various/specific components involved in the cellular responses to mold [28,29]. More specifically, CLRs like dectin-1 and dectin-2 have been suggested to play a central role in different part of the antifungal defense [30]. Different part of the mold seems to trigger reactions via different receptors and receptors may interact to mediate various types of immune responses [28]. Ligand binding to these receptors trigger activation of the transcription factor Nuclear Factor (NF)-κB, a central regulator of pro-inflammatory responses [31,32]. Less is known with regard to the relative role of TLR2 versus TLR4 and how they regulate specific pro-inflammatory responses due to the outer surface components found on aerosolized spores and hyphal fragments. There are especially few studies conducted in lung epithelial cells.

We have recently provided a comprehensive characterization of X-ray treated (aerosolized) spores and hyphal fragments from several mold species with respect to morphological, chemical and biological properties [33]. Their pro-inflammatory responses have also been investigated in various macrophage models (Øya et al., submitted). The aim of the present study was to further characterize and compare the cytokine/chemokine responses of spores and hyphal fragments from various mold species in human bronchial epithelial cells (BEAS-2B). Most interestingly, while X-ray treated spores did not induce pro-inflammatory responses, hyphal fragments of both the pathogenic *A. fumigatus* and the far less pathogenic *P. chrysogenum* enhanced a TLR2-dependent expression and release of IL-6 and IL-8.

## 2. Materials and Methods

### 2.1. Chemicals

LHC-9 cell culture medium was from Invitrogen (Carlsbad, CA, USA) and PureCol™ collagen from Inamed Biomaterials (Fremont, CA, USA). Cytokine ELISA assay for TNF-α (Human TNF-α Cytoset), IL-6 (Human IL-6 Cytoset) and CXCL8 (Human IL-8 Cytoset) were purchased from Life Technologies (Camarillo, CA, USA). Cytokine ELISA assay for IL-1β (Human IL-1β Duoset), human TLR2 antibody (MAB2616) and human TLR4 antibody (AF1478) from R&D Systems, Inc. (Minneapolis, MN, USA). NukleoSpin RNA Plus was purchased from Macherey-Nagel (Dürer, Germany) and High Capacity cDNA Archive Kit, TaqMan Universal PCR Mastermix and TaqMan Gene Expression Assay were purchased from Applied Biosystems (Foster City, CA, USA). Propidium iodide (PI) and Hoechst 33342 (Hoechst) were purchased from Sigma-Aldrich Chemical Company (St Louis, MO, USA). All other chemicals used were purchased from commercial sources at the highest purity available.

### 2.2. Cell Culture and Treatments

BEAS-2B cells, which is a SV-40 hybrid (Ad12SV40) transformed human bronchial epithelial cell line, was purchased from European Collection of Cell Cultures (ECACC, Salisbury, UK). Cells were maintained in serum-free LHC-9 medium on collagen-coated flasks in a humidified atmosphere at 37 °C with 5% CO_2_, passaged twice per week with refreshment of medium every second day. Two days prior to exposure, cells were plated into collagen-coated 35 mm 6-well culture dishes (2.3 × 10^5^ cells/well in 1 mL) or 10 mm 12-well culture dishes (8 × 10^4^ cells/well in 0.5 mL) and grown to near confluence. In all experiments medium was changed the day after seeding and before exposure.

Cells were exposed to X-ray- or untreated preparations of spores and hyphal fragments of mold at various concentrations and for different time duration. The highest concentration used 100 µg dry weight/mL for *A. fumigatus* corresponding to approximately 3 × 10^6^ spores. In order to investigate TLR2 and TLR4 involvement in cytokine/chemokine gene expression and release, cells were pretreated for 1 h with specific monoclonal antibodies (mAbs) against TLR2 or TLR4 and subsequently exposed to hyphae of *A. fumigatus* or *P. chrysogenum* for 6 h.

### 2.3. Preparation of Mold Samples

Three types of fungal samples prepared from four pure fungal species isolates were tested, and representative isolate used in this study. Spores collected by washing the culture (washed spores; ws), spores collected by air flow over the culture (aerosolized spores; as), and mycelial mass collected by cyclone-vacuuming freeze dried and grinded mycelial mass harvested prior to sporulation (hyphal fragments; hf). The methods used for preparation and their characterization have been previously reported [33]. In short, isolates comprised *A. fumigatus* Fresenius 1863 (strain A1258 FGSC) purchased from the Fungal Genetics Stock Center (University of Missouri, Kansas City, KS, USA), *A. versicolor* Tirobaschi 1908 (strain VI 03554), *P. chrysogenum* Thom 1910 (strain VI 04528), and *S. chartarum* (Ehrenb) S. Hughes (VI 03618) obtained from the Section of Mycology at the Norwegian Veterinary Institute (Oslo, Norway). Specific mold was grown on agar covered with cellophane membrane. The mold preparations were washed to remove enzymes/mycotoxins (to prohibit their interference with the observed pro-inflammatory responses) and inactivated/“killed” on ice with X-ray (17,45 Gy/min, 225 kV, 13 mA, no filter, 5 cm distance from the source) from X-RAD 225 (Precision X-ray Inc., North Branford, CT, USA) receiving a total dose of 5 kGy.

### 2.4. Cytokine/Chemokine Release

Pro-inflammatory cytokines were analyzed in the supernatants after exposures using quantitative sandwich enzyme-linked immunosorbent assay (ELISA) kit. The kits were performed according to the manufacturer’s standard protocols. The corresponding limits of detection were as follows: IL-6 (CHC 1263); 15.6 pg/mL, IL-8 (CHC 1303); 12.5 pg/mL, TNF-α (CHC1753), 15.6 pg/mL, IL-1α (DY200); 7.81 pg/mL and IL-1β (DY201); 3.9 pg/mL. Absorbance was quantified using a plate reader (TECAN Sunrise, Phoenix Research Products, Hayward, CA, USA) equipped with a dedicated software (Magellan V1.10; TECAN Austria GmbH, Grödig-Salzburg, Austria).

### 2.5. Gene Expression

Total RNA was isolated using NukleoSpin RNA Plus (Macherey-Nagel) and reverse transcribed to cDNA on a PCR System 2400 (PerkinElmer, Waltham, MA, USA), using a High Capacity cDNA Archive Kit (Applied Biosystems). Real-time PCR was performed using pre-designed TaqMan Gene Expression Assays and TaqMan Universal PCR Master Mix and run on Applied Biosystems 7500 fast software. Gene expressions of induced IL-1α (Hs00174092_m1), IL-1β (Hs01555410_m1), TNF (Hs01113624_g1), IL-6, (Hs00174131_m1), IL-8, (Hs00174103_m1) and IL-33 (Hs00369211_m1) were normalized against ACTB (Hs01060665_g1), and expressed as fold change compared to untreated control as calculated by the ΔΔCt method (ΔCt = Ct[Gene of Interest]—Ct[ACTB]; ΔΔCt = ΔCt[Treated]—ΔCt[Control]; Fold change = 2^[−ΔΔCt]^).

### 2.6. Microscopic Examination of Germinative Potential

Viable *A. fumigatus* airborne spores were incubated in LHC-9 medium in collagen-coated 24-well culture dishes on BEAS-2B cells or without cells. The germinative potential was examined after 24 h exposure with a digital camera (Nikon D40, Nikon Corp., Japan) coupled to a light microscope.

### 2.7. Cytotoxicity

After exposure, the cell morphology of the cultures was visually examined with a light microscope. Toxicity was assessed and categorized as the relative amount of floating cells (dead cells) in the cultures versus attached cells (living cells). Cell shape and vacuoles were recognized and documented. Cells were examined after exposures and their viability was determined using fluorescence microscopy after staining with Propidium iodide (PI) and Hoechst 33342 [33]. At least 400 cells were counted per sample.

### 2.8. Statistical Analysis

**Figure 1:**
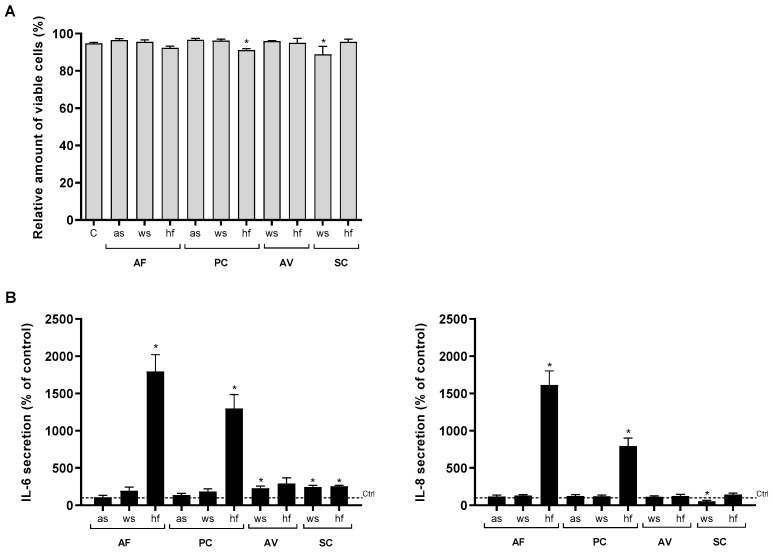
Cell viability and cytokine/chemokine release after exposure to spores and hyphal fragments from various mold species. BEAS-2B cells were exposed to 100 µg dry weight/mL of X-ray treated aerosolized spores (as), washed spores (ws) or hyphal fragments (hf) from *A. fumigatus* (AF), *P. chrysogenum* (PC), *A. versicolor* (AV), *S. chararum* (SC) or medium (control) for 6 h, and assessed for: (**A**) cytotoxicity by staining with PI and Hoechst and analyzed with fluorescence microscopy. The results represent mean ± SEM of >3 independent experiments. Statistical analyses were performed by one-way ANOVA with Dunnett’s post-tests on log transformed data. Significant difference compared to the control denoted by * *p* < 0.05, or (**B**) IL-6 and IL-8 release measured by ELISA. Bars represent mean ± SEM concentrations of >6 independent experiments. Statistical analyses were performed by two-way ANOVA with Dunnett’s post-tests on 2-step normalized data. Significant difference compared to the control denoted by * *p* < 0.05.

All statistical analyses were performed in GraphPad Prism 7 (GraphPad Software Inc., La Jolla, CA, USA). Two-way analysis of variance (ANOVA) with Dunnett post-test was used to analyze the data sets. As specified in the figure legends, some data were log transformed before performing ANOVA to fulfil the assumption of homogeneity of variance in the data sets. We performed a standard deviation vs. mean test to evaluate if log transformation of data was needed. The data in Figures 1B, 5 and 7B were normalized using a two-step procedure to scale the controls (Figures 1B and 7B) or maximum response level (Figure 5) within in each data set to the same level while still retaining variation, allowing for ANOVA analysis [34]. This was done due to large variations in the basal response levels between different experiments. In short, the data from each experiment was first normalized to the mean of all data in the experiment. Then, the data in each data set, consisting of >3 different experiments, were normalized to the mean of the controls or maximum responses. Results are expressed as mean ± standard error of mean (SEM). *p* values less than 0.05 were considered significant.

## 3. Results

### 3.1. Cytokine/Chemokine Release after Exposure to the Different Mold Species

The isolate of *A. versicolor* and *S. charatum* used in this study do not form airborne spores in sufficient quantities. Thus, two different preparations of *A. fumigatus* and *P. chrysogenum* spores were tested: spores collected by airflow (aerosolized spores; as) and by washing the culture (washed spores; ws) as well as their hyphal fragment preparations, to allow for comparison with the spores from *A. versicolor* and *S. charatum*.

First, we characterized the cytotoxicity of the various X-ray treated mold samples in the BEAS-2B cells. As judged by light microscopy examining cell density, morphology and floating cells, neither of the mold preparations appeared to affect cell viability after 6 or 24 h at the concentration tested (not quantified). This was supported by fluorescence microscopy following staining with PI/Hoechst to identify specifically apoptotic as well as necrotic cells, showing no marked increase in cytotoxicity after 6 h (Figure 1A) and 24 h (data not shown).

Next, we investigated their relative potential to release cytokine/chemokine in BEAS-2B. Whereas the spore preparations of *A. fumigatus* and *P. chrysogenum* had little or no inflammatory potential, their hyphal fragments (hf) both induced a marked release of IL-6 and IL-8, with *A. fumigatus* being slightly more potent. In contrast, spores from *A. versicolor* and *S. chartarum* triggered some minor increase in IL-6, and hyphal fragments from *S. chartarum* a slight increase in IL-6 release (Figure 1B). Enhanced IL-1α, IL-1β and TNF-α release were not detected after exposure to any of the mold preparations (data not shown). Whereas *A. fumigatus* and *P. chrysogenum* hyphal fragments in general were more potent than their respective spore fractions, responses induced by both spores and hyphal preparations of *A. versicolor* and *S. chartarum* were rather similar (Figure 1B).

For the following experiments we decided to compare the two most pro-inflammatory species, *A. fumigatus* and *P. chrysogenum* including their spores and hyphal fragments collected by air flow as they are more relevant for inhalation exposure.

### 3.2. Role of Experimental Conditions

**Figure 2:**
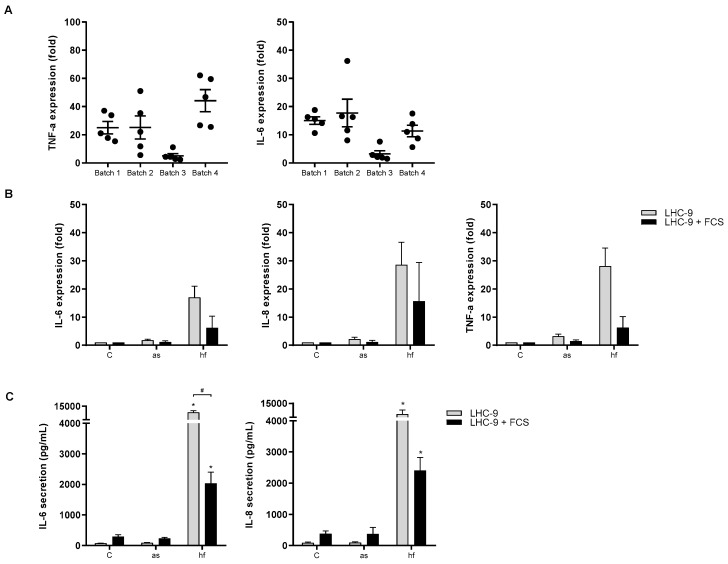
Batch variations and effects of serum on cytokine/chemokine expression and release. (**A**) BEAS-2B cells were exposed to 100 µg dry weight/mL of four different batches of X-ray treated *P. chrysogenum* hyphal fragments (hf) for 6 h. The mRNA level of TNF-α and IL-6 were assessed by real time RT-PCR. Medium blanks were included as control. Plots represent mean ± SEM of 5 independent experiments. (**B**) BEAS-2B cells grown in LHC-9 media ± FCS were exposed to X-ray treated aerosolized spores (as) and hyphal fragments (hf) 100 µg dry weight/mL from *P. chrysogenum* for 6 h and assessed for expression with real-time RT-PCR or (**C**) cytokine/chemokine release analyzed by ELISA. Medium blanks were included as control. The results represent mean ± SEM of two or three independent experiments respectively. Statistical analyses were performed by two-way ANOVA with Dunnett’s/Sidak post-tests on log transformed data. Significant difference denoted by * (individual control vs exposed) or ^#^ (±FCS), *p* < 0.05.

To ensure that the difference in pro-inflammatory responses induced by the different mold species/growth stages were not due to coincidence, the cytokine responses induced by four different mold preparations (batches) were examined. Here we present only the data obtained with hyphal fragments from *P. chrysogenum*, but the variability were representative for the results obtained from all preparations. As seen in Figure 2A, the variation in TNF-α and IL-6 responses between three different preparations of hyphal fragments were more or less in the same range as that seen between experiments with hyphal fragments from the same batch, whereas one batch deviated from the rest. In the rest of the experiments we compared responses of all mold samples in the same experiment with batches giving representative responses (Figure 2A). Thus, the possibility that the small differences in responses induced by hyphal fragments from *A. fumigatus* and *P. chrysogenum* were merely due to batch-variations cannot be excluded.

Compared to responses observed in macrophages (Øya et al., submitted), the responses to spores and hyphal fragments in epithelial cells were very low. As BEAS-2B cells, in contrast to macrophages, were grown in serum-free medium we explored if addition of serum would enhance the epithelial responses to spores and hyphal fragments. However, addition of fetal calf serum (FCS) rather reduced both the expression of IL-6, IL-8 and TNF-α (Figure 2B) as well as the release of IL-6 and IL-8 (Figure 2C) after exposure to hyphal fragments of *P. chrysogenum*.

### 3.3. Concentration-Dependent Release of IL-6 and IL-8 after Exposure to A. fumigatus and P. chrysogenum

**Figure 3:**
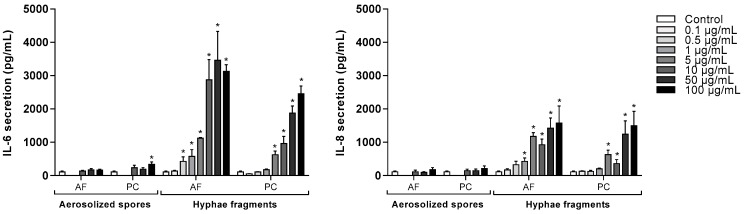
Release of IL-6 and IL-8 from BEAS-2B after exposure to spores and hyphal fragments from *A. fumigatus* and *P. chrysogenum*. Cells were exposed to different concentrations of X-ray treated aerosolized spores, hyphal fragments or medium (controls) for 6 h. Release of IL-6 and IL-8 were measured by ELISA. Bars represent mean ± SEM of 3–7 independent experiments. Statistical analyses were performed by two-way ANOVA with Dunnett’s post-tests on log transformed data. Significant difference compared to the control denoted by * *p* < 0.05.

To examine the pro-inflammatory effects of *A. fumigatus* and *P. chrysogenum* aerosolized spores and hyphal fragments in more detail, BEAS-2B cells were exposed to various concentrations for 6 h. Both hyphal preparations increased the release of IL-6 and IL-8 in a concentration-dependent manner, but in support of our initial results; *A. fumigatus* appeared to be slightly more potent than *P. chrysogenum*. *A. fumigatus* and *P. chrysogenum* hyphae-induced IL-6 responses started at 0.5 and 5 µg dry weight/mL, and IL-8 release at 1 and 5 µg dry weight/mL, respectively (Figure 3). Only aerosolized *P. chrysogenum* spores at the highest concentration tested (100 µg dry weight/mL) induced a statistically significant increase in IL-6 release, but the effects were marginal compared to the effects of hyphal fragments. No marked effects on IL-8 release were observed in spore-exposed cells (Figure 3). Furthermore, neither spores nor hyphal fragments from the two species induced IL-1α, IL-1β or TNF-α release (data not shown).

### 3.4. Gene Expression after Exposure of A. fumigatus and P. chrysogenum

**Figure 4:**
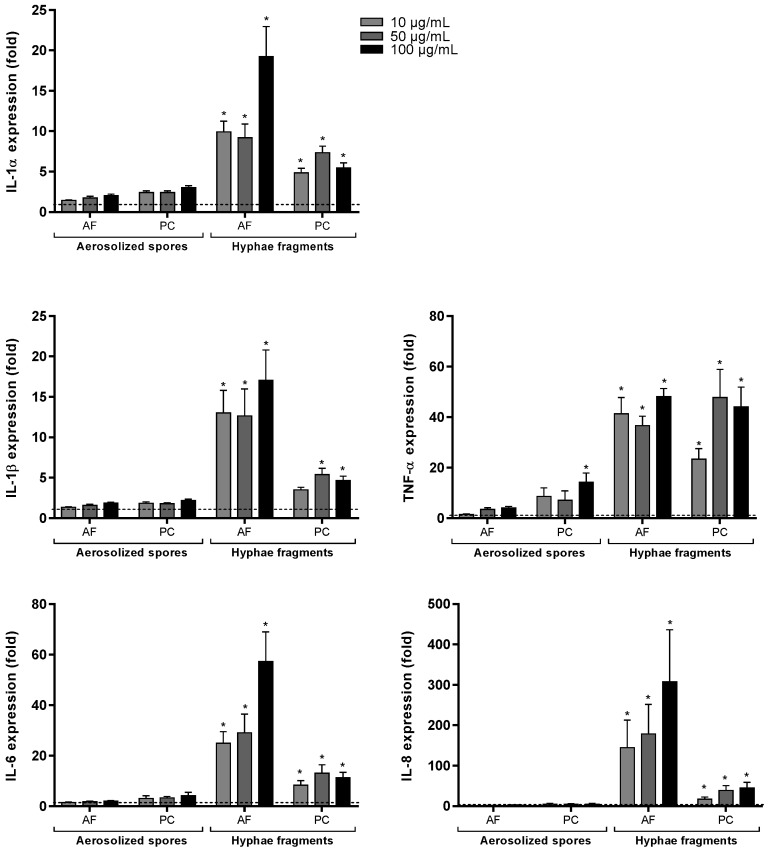
Expression of cytokines/chemokines in BEAS-2B after exposure to spores and hyphal fragments from *A. fumigatus* and *P. chrysogenum*. Cells were exposed to three different concentrations (10, 50 and 100 µg dry weight/mL) of X-ray treated aerosolized spores and hyphal fragments for 6 h. Medium blanks were included as control. Expression of IL-α, IL-1β, TNF-α, IL-6 and IL-8 were measured with real-time RT-PCR. Bars represent mean ± SEM of 4–5 independent experiments. Statistical analyses were performed by two-way ANOVA with Dunnett’s post-tests on log transformed data. Significant difference compared to the control denoted by * *p* < 0.05.

The gene expression of various cytokines/chemokines in BEAS-2B cells were also measured after 6 h exposure to *A. fumigatus* and *P. chrysogenum* preparations. As seen in Figure 4, hyphal fragments of both species significantly upregulated IL-1α, IL-1β, TNF-α, IL-6 and IL-8 expression at the concentrations tested. Except for TNF-α, *P. chrysogenum* hyphal fragments induced less response than *A. fumigatus*. In line with previous findings on protein secretion, hyphal fragments induced a stronger response than spores. Only *P. chrysogenum* aerosolized spores elicited a weak but statistically significant increase in the expression of TNF-α at the highest concentration (Figure 4).

### 3.5. The Role of TLR2 and TLR4 in A. fumigatus and P. chrysogenum Hyphae-Induced Release of IL-6 and IL-8

**Figure 5:**
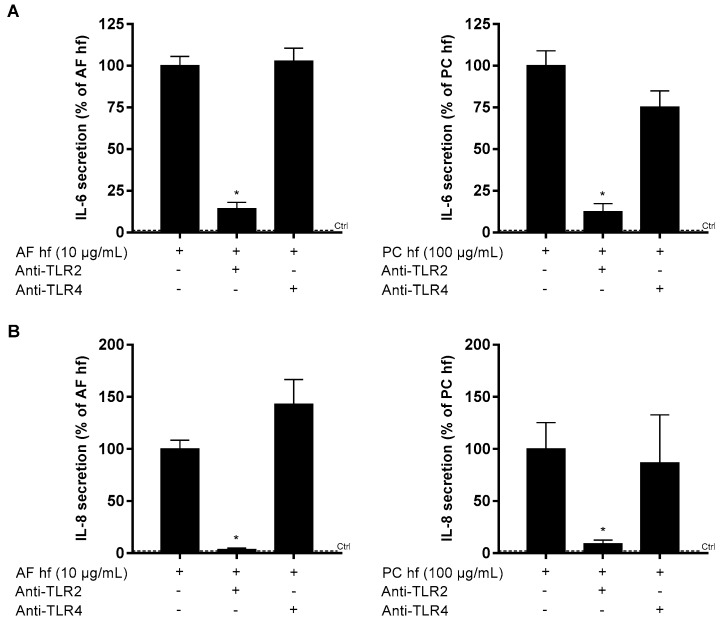
Inhibition of *A. fumigatus*- or *P. chrysogenum*-induced IL-6 and IL-8 release by attenuating TLR2 and TLR4. BEAS-2B cells were pretreated with human TLR2 blocking antibody MAB2616 (0.2 µg/mL) or TLR4 antagonist AF1478 (10 µg/mL) for one h and further incubated with X-ray treated hyphal fragments (hf) of *A. fumigatus* (AF; 10 µg dry weight/mL) or *P. chrysogenum* (PC; 100 µg dry weight/mL) for 6 h. Medium blanks were included as control. Release of (**A**) IL-6 and (**B**) IL-8 were assessed by ELISA. Bars represent mean ± SEM of three independent experiments. Statistical analyses were performed by two-way ANOVA with Dunnett’s post-tests on 2-step normalized data. Significant difference compared to the respective hyphal fragments denoted by * *p* < 0.05.

Pretreatment with TLR2 blocking antibodies reduced IL-6 and IL-8 release induced by both *A. fumigatus* and *P. chrysogenum* hyphal fragments in BEAS-2B cells. The inhibitory effect was most pronounced for IL-8, which was almost completely abrogated by antibody treatment. In contrast, no statistical significant effects on IL-6 and IL-8 were observed after TLR4 antagonist treatment (Figure 5). Similar observations were seen when examining the effects of TLR2-antagonist on IL-6 and IL-8 expression level. Notably, these studies also indicated that the increased expression of IL-1β, TNF-α were linked to TLR2 (Supplementary Figure S1).

### 3.6. Effects of Untreated A. fumigatus Spores on the Gene Expression and Release of IL-6 and IL-8

**Figure 6:**
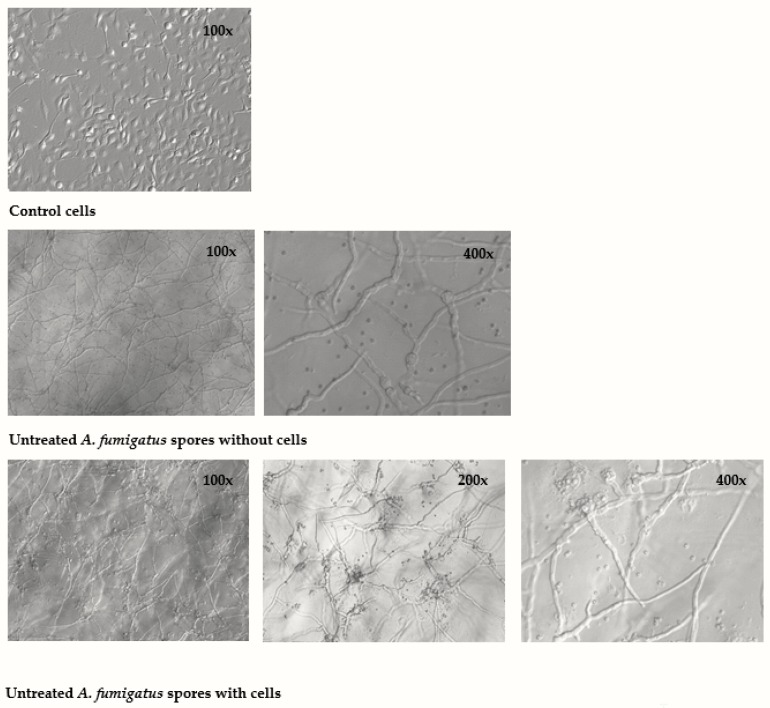
Germinative potential of untreated spores of *A. fumigatus*. Untreated *A. fumigatus* aerosolized spores (10 µg dry weight/mL) were added to cell culture dishes without or with BEAS-2B cells and incubated for 24 h. Medium blanks were included as control. The potential to form hyphae and spores were analyzed by light microscopic pictures with original magnification 100, 200 or 400×.

Finally, we wanted to compare the effect of untreated (viable) mold spores in BEAS-2B cells, with the results obtained by exposure to X-ray treated (nonviable) aerosolized spores and hyphal fragments samples. In order to examine if BEAS-2B cells influenced the capacity of spores to form hyphae, untreated *A. fumigatus* and *P. chrysogenum* aerosolized spores were added to dishes with or without BEAS-2B cells for 24 h. As shown in Figure 6, *A. fumigatus* formed hyphae in presence and absence of BEAS-2B cells. Hyphae formation by *A. fumigatus* spores could be observed already after 9 h (data not shown). After 24 h, the mold totally covered the BEAS-2B cells, which by microscopic examination seemed to be dead. Interestingly, new spores were evident around hyphae bud in the dish with BEAS-2B cells, whereas no new spores where formed without cells (400× magnification in Figure 6). By contrast, untreated *P. chrysogenum* aerosolized spores germinated poorly and appeared to be unable to form hyphae (data not shown).

**Figure 7:**
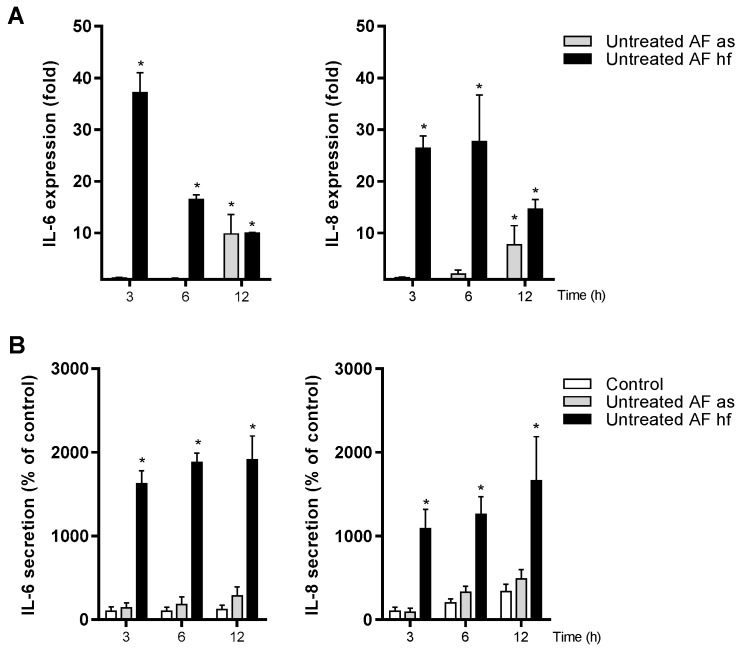
Time-dependent expression and release of IL-6 and IL-8 after exposure to untreated spores and hyphal fragments from *A. fumigatus*. Cells were exposed to untreated aerosolized spores (as) or hyphal fragments (hf) of *A. fumigatus* (67 µg dry weight/mL) for three different time points. Medium blanks were included as control. (**A**) Expression of IL-6 and IL-8 were measured by real-time RT-PCR. (**B**) Release of IL-6 and IL-8 were measured with ELISA. Bars represent mean ± SEM of <4 independent experiments. Statistical analyses were performed by two-way ANOVA with Dunnett’s post-tests on log transformed or 2-step normalized data respectively. Significant difference compared to the control denoted by * *p* < 0.05.

The untreated *A. fumigatus* spores increased the expression of IL-6 and IL-8 after 12 h exposure (Figure 7A). In contrast, statistical significant increased release of these two markers was not observed at this time point (Figure 7B).

Preliminary experiments suggested that untreated spores more rapidly increased gene expression of IL-1β and TNF-α (Supplementary Figure S2) than IL-6 and IL-8 (Figure 7A). Interestingly, in these experiments we also found an increase in IL-33 (Supplementary Figure S2), another alarmin/cytokine from the IL-1 family [35]. Furthermore, X-ray treated spores and hyphal fragments also increased the expression of IL-1β and TNF-α, as well as IL-6, IL-8 and IL-33 in a time-dependent manner (Supplementary Figure S3). However, the spore-induced response was delayed and low when compared to that of hyphal fragments.

## 4. Discussion

This study showed that X-ray treated hyphal fragments from pathogenic *A. fumigatus* and less pathogenic *P. chrysogenum* induced synthesis and release of IL-6 and IL-8 from BEAS-2B cells. In contrast, their treated spores triggered marginal effects. Minor effects were seen after exposure to spore and hyphal preparations from *A. versicolor* and *S. chartarum*. Notably, hyphal fragments from *A. fumigatus* and *P. chrysogenum* also increased gene-expression, but not the release, of IL-1α, IL-1β and TNF-α. Furthermore, TLR2 seemed to be involved in both *A. fumigatus* and *P. chrysogenum* hyphae-induced IL-6 and IL-8 release in BEAS-2B, in contrast to previous studies in macrophages pointing toward a role of TLR4 (Øya et al., submitted).

*A. fumigatus* is a well-known mold pathogen and induce pro-inflammatory reactions which are considered important for host defense [32]. Thus, *A. fumigatus* is an often used model in studies of cellular recognition and mechanisms involved in various inflammatory responses triggered by mold. Less is known with regard to the relative pro-inflammatory responses of other fungal species. In the present study, we found large variation in the cellular cytokine/chemokine responses after exposing BEAS-2B cells to hyphal fragments of different mold species. The highest pro-inflammatory responses and effects at the lowest concentrations were induced by hyphal preparations from *A. fumigatus* and *P. chrysogenum*. This resembles previous observations in various macrophage models (Øya et al., submitted).

Compared to the epithelial cell results, substantially higher cytokine/chemokine responses were observed in macrophages (Øya et al., submitted).These enhanced responses did not appear to be related to enhanced recognition due to serum proteins in the macrophage medium. The addition of serum to the BEAS-2B medium rather reduced the responses in these cells, suggesting that the serum proteins rather blocked epitopes important for the pro-inflammatory effects. In accordance with this, serum proteins are known to inhibit the reactivity of mineral particles by blocking active sites on their surface, thus inhibiting interaction of particles with cell surface receptors or membrane lipids [36,37].

As pathogens like *A. fumigatus* have been reported to escape recognition by the immune system [38], we hypothesized that the less pathogenic species induced more pro-inflammatory responses. However, there was no apparent correlation between the known pathogenicity of the mold species and their pro-inflammatory effects. In fact the hyphae stage of the more pathogenic *A. fumigatus* was at least as potent as the far less pathogenic *P. chrysogenum,* and much more potent than the two other species tested. In addition, studies have demonstrated considerably higher levels of mold fragments than of intact spores in mold-contaminated buildings [6,39]. Taken into account high prevalence of *P. chrysogenum* [24], the high pro-inflammatory effects found could indicate that mold fragments of this species may be candidate for chronic pro-inflammatory diseases associated with mold exposure.

We further show that hyphae from *A. fumigatus* and *P. chrysogenum* induced much higher IL-6 and IL-8 responses, and induced effects at much lower concentrations than their respectively spore preparation. This difference between growth stages is not necessarily found with all mold species, as our results show that X-ray treated spores and hyphal fragments from both *A. versicolor* and *S. chartarum* gave rather similar, although low, responses.

Interestingly, Beisswenger et al. [40] have reported that differentiated primary human respiratory epithelial cells (HBEC) recognize inactivated (heat or UV treated) resting *A. fumigatus* spores, but not swollen spores or hyphae. Furthermore, resting spores triggered induction of the interferon (IFN)-β signaling pathway. Expression of IFN-β-inducible genes were enhanced, including IFN-γ-inducible protein (IP)-10, which plays an important role in directing immune cells to the respiratory tract to ensure efficient clearance of inhaled spores. The contrast between these interesting findings and our present results, may relate to differences in the experimental approach (lower amount of spores), cell type dependent effects, as well as differences in the inflammatory markers measured. It should be noted that macrophages seem to be more sensitive to X-ray treated spores (Øya et al., submitted) compared to lung epithelial cells, supporting that the observed response patterns are cell type dependent.

IL-1 family members and TNF-α plays central roles in onset of both the innate and adaptive immune system but are typically produced as pro-forms and require proteolytic processing in order to unlock their full biological potential [41]. For instance, pro-IL-1β requires cleavage by activated caspase-1 in order to be released in its active form [42]. Similarly pro-IL-1α require cleavage by calpain in order to be released [43], while TNF-α is processed in a membrane-bound pro-form which is cleaved and released by TNF-α converting enzyme (TACE/ADAM17) [41]. Without activation of this essential cleavage step, neither IL-lα, IL-1β, IL-33 nor TNF-α could be released from living cells. However, un-cleaved pro-IL-lα and pro-IL-33 are biologically active and acts as alarmins when released from necrotic cells, for instance at the site of barrier breaching [44]. 

Although exposure to hyphal fragments from *A. fumigatus* and *P. chrysogenum* did not increase release of the pro-inflammatory cytokines IL-lα, IL-1β and TNF-α in BEAS-2B, they markedly increased cytokine gene expression. Also the expression of IL-33 was increased. This increased gene expression in absence of protein release, may indicate increased synthesis of pro-forms without a following activation of the cleavage step required for release of these cytokines. It is therefore tempting to speculate that the secondary activation of caspase-1, calpain and TACE was not initiated by hyphal fragments or spores. In this aspect hyphal fragments and spores appear to resemble bacterial lipopolysaccharide, which is known to restrictively activate IL-1β gene expression and synthesis of pro-IL-1β, but not the caspase-1 activation required for release of mature IL-1β [45]. In contrast to BEAS-2B, *A. fumigatus* and *P. chrysogenum* hyphal fragments increased both the synthesis and release of IL-1β and TNFα from macrophages (Øya et al., submitted). Thus suggesting that the expression of receptors/enzymes and/or multiprotein complex involved in their proteolytic processing are differentially expressed/activated in the two cell types.

Importantly, increased expression of these cytokines/alarmins may have implications even if not released, as their proteolytic processing may be triggered by other agents [45]. More specifically, co-exposure of *A. fumigatus* hyphal fragments and the mineral particle quartz resulted in synergistic releases of IL-1β due to an activation of the inflammasome [45]. The un-cleaved pro-forms of IL-1α and pro-IL-33 also display basal activity when released by necrotic cells [46]. Furthermore, membrane-bound pro-TNF-α also displays biological activity, although its effect may differ from soluble TNFα [47].

The role of specific receptors in mold recognition and inflammatory responses are mostly studied in various types of immune cells using spores, crude aqueous extracts and/or isolated single components [29,48,49,50]. There are studies suggesting that untreated *A. fumigatus* spores induce Dectin-1 receptor in HBE cells in a TLR2-dependent manner, and are involved in the expression of TNF-α, GM-CSF and IL-8 [51]. The relative roles of TLR2 and TLR4 in the active defense against pathogenic *A. fumigatus* versus less pathogenic fungal species such as *P. chrysogenum* in lung epithelial cells have to our knowledge so far not been studied. Furthermore, the samples used were well-characterized X-ray treated aerosolized spore and hyphal fragments that were washed to prohibit toxic effects/interference of mycotoxins and soluble proteases. Thus, our findings specifically reveal the role of outer surface components/structures found on aerosolized spores and hyphal fragments [33].

Various TLRs may interact with other TLRs as well as Dectin-1 or adapter/accessory proteins like MyD88, CD14 and MD-2 to mediate immune responses to mold [28,52,53]. We found that TLR2 seem to be involved in the expression and release of IL-6 and IL-8 triggered by hyphal fragments of the pathogenic *A. fumigatus* as well the less pathogenic *P. chrysogenum* in BEAS-2B cells. Furthermore, preliminary experiments suggest that hyphal fragments of *A. fumigatus* and *P. chrysogenum* also regulate the expression of IL-1α, IL-1β and TNF-α by mechanisms involving TLR2. Another study on BEAS-2B cells report that germinating *A. fumigatus*-induced IL-8 synthesis was controlled by the phosphatidylinositol 3-kinase (PI3K), p38 mitogen activated protein kinase (MAPK), and ERK1/2 pathways, independent of the TLR/MyD88 [54]. This indicate that living hyphae secrete proteins not included in our preparations, which may give pro-inflammatory responses. Our results in BEAS-2B is also in contrast to our recent findings in human blood monocyte-derived macrophages (MDM), where both *A. fumigatus* spores and hyphae seemed to induce IL-1β, TNF-α, IL-6 and IL-8 release depending on TLR4 rather than TLR2 (Øya et al., submitted). Although we have not specifically tested the degree of receptor inhibition, results with BEAS-2B cells show that the TLR2 blocking antibody almost completely inhibited the release of IL-8, whereas the TLR4 inhibitor reduced the IL-6 response by 75% in macrophages (Øya et al., submitted).

In macrophages, TLRs are often expressed at the cell surface [55,56], while in bronchial epithelial cells these receptors tend to be located intracellularly [57,58,59]. These findings are in accordance with our findings of higher pro-inflammatory potential in different macrophage models (Øya et al., submitted). Possibly there may also be cell type differences with regard to the relative amount of TLR2 versus TLR4 expression that might explain their highly different pro-inflammatory role in the two cell types. Intracellular localization of TLRs may also interfere with the degree of inhibition by the TLR2/4-inhibitor, thus these studies should be followed up in cells using siRNA and/or CRISPR/CAS9 technology. As the lung is constantly exposed to potentially inflammatory components, the intracellular localization of TLRs in lung epithelium may play an important role in the prevention of inappropriate/deleterious activation of immune responses that can cause development of chronic inflammatory disease.

Additionally, we found that exposure to untreated spores from *A. fumigatus* in BEAS-2B cells induced a cytokines/chemokine profile with similarities to hyphal fragments. As judged from hyphal filament formation, co-culture of *A. fumigatus* and BEAS-2B cells did not reduce the spores’ capacity to germinate. As the BEAS-2B cells appeared to be killed at the time studied, the cells rather seemed to function as a positive substrate that stimulated new sporulation in the present study. Interestingly, in strong contrast to the pathogenic *A. fumigatus*, spores of *P. chrysogenum* germinated poorly.

## 5. Conclusions

In conclusion, X-ray treated hyphal fragments from both pathogenic *A. fumigatus* and less pathogenic *P. chrysogenum* induced synthesis and release of IL-6 and IL-8 from BEAS-2B cells via TLR2. An increased gene expression of IL-1α, IL-1β and TNF-α were also seen. The high pro-inflammatory potential of *P. chrysogenum* hyphal fragments combined with its high prevalence in damp indoor environments may be of importance with regard to respiratory diseases linked to chronic pro-inflammatory responses.

## Supplementary Materials

The following are available online at https://www.mdpi.com/1660-4601/16/6/1085/s1, Figure S1: Inhibition of *A. fumigatus*- or *P. chrysogenum*-induced cytokine/chemokine gene expression by TLR2 and TLR4 antagonist molecules. Figure S2: Time-dependent IL-1β, TNF-α and IL-33 expression after exposure to untreated *A. fumigatus* spores and hyphal fragments. Figure S3: Time-dependent gene expression after exposure to X-ray treated *A. fumigatus* spores and hyphal fragments.
