# Supplementary material for: Pro-Inflammatory Responses in Human Bronchial Epithelial Cells Induced by Spores and Hyphal Fragments of Common Damp Indoor Molds
Source: Int J Environ Res Public Health. 2019 Mar 26;16(6):1085. doi: 10.3390/ijerph16061085 (PMC6466608; doi:10.3390/ijerph16061085)
Supplement: Supplementary file 1 [file ijerph-16-01085-s001.pdf]

International Journal of
Environmental Research and Public Health

Supplementary

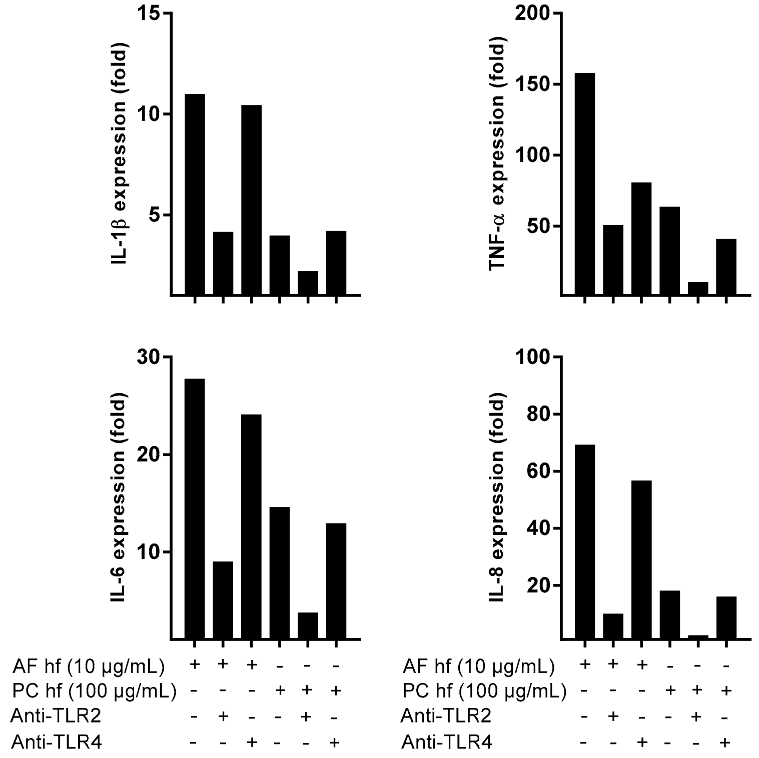

Supplementary Figure S1: Inhibition of ***A.*** *fumigatus*- or *P. chrysogenum*-induced cytokine/chemokine gene expression by TLR2 and TLR4 antagonist molecules. Cells were pretreated with human anti-TLR2 (MAB2616, 0.2 µg/mL) or anti-TLR4 (AF1478, 10 µg/mL) for 1 h and further incubated with X-ray treated hyphal fragments (hf) of *A. fumigatus* (AF; 10 µg/mL) or *P. chrysogenum* (PC; 100 µg/mL) for 6 h. Medium blanks were included as control. The expression of IL-1β, TNF-α, IL-6 and IL-8 were assessed with real-time RT-PCR. Bars represent one independent experiment.

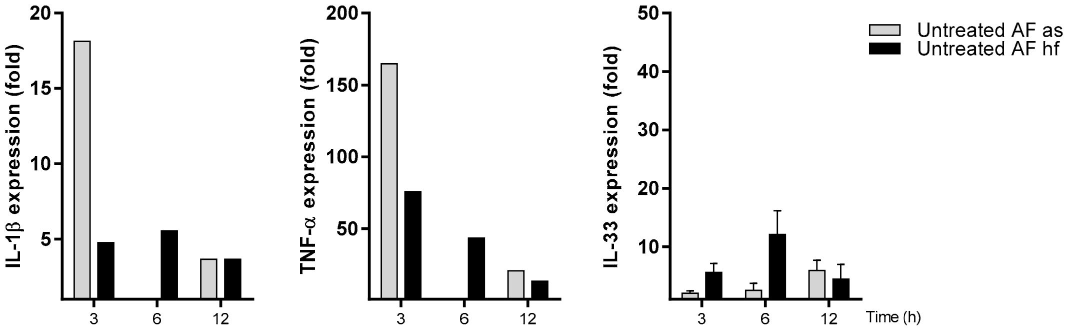

Supplementary Figure S2: Time-dependent IL-1β, TNF-α and IL-33 expression after exposure to untreated ***A.*** *fumigatus* spores and hyphal fragments. Cells were exposed to *A. fumigatus* aerosolized spores (as), hyphal fragments (hf) for three different time points. Medium blanks were included as control. The expression of IL-1β, TNF-α and IL-33 were assessed by real-time RT-PCR. Bars represent one independent experiment or the mean ± SEM of three independent experiments (IL-33).

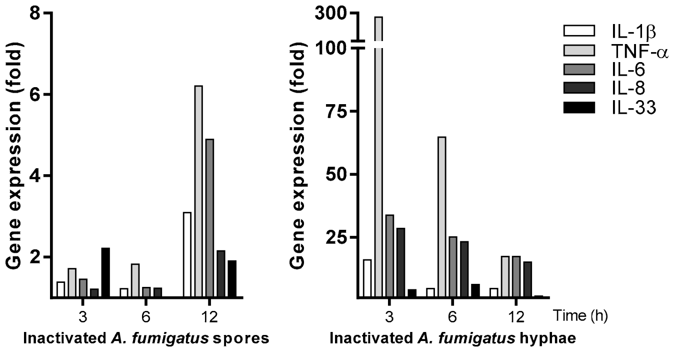

Supplementary Figure S3: Time-dependent gene expression after exposure to X-ray treated *A. fumigatus* spores and hyphal fragments. Cells were exposed to X-ray treated *A. fumigatus* aerosolized spores (as), hyphal fragments (hf) for three different time points. The expression of IL-1β, TNF-*α*, IL-6, IL-8 and IL-33 were assessed by real-time RT-PCR. Bars represent one independent experiment.
